# The Cancer-Associated Antigens Sialyl Lewis^a/x^ and Sd^a^: Two Opposite Faces of Terminal Glycosylation

**DOI:** 10.3390/cancers13215273

**Published:** 2021-10-21

**Authors:** Fabio Dall’Olio, Michela Pucci, Nadia Malagolini

**Affiliations:** Department of Experimental, Diagnostic and Specialty Medicine (DIMES), General Pathology Building, University of Bologna, Via San Giacomo 14, 40126 Bologna, Italy; michela.pucci3@unibo.it (M.P.); nadia.malagolini@unibo.it (N.M.)

**Keywords:** glycosylation, colorectal cancer, Sd^a^ antigen, Sialyl Lewis antigens, glycosyltransferases, *B4GALNT2*, gene expression control, transcriptomic analysis

## Abstract

**Simple Summary:**

The glycosyltransferase β1,4-N-acetylgalactosaminyltransferae 2 (*B4GALNT2*), product of the *B4GALNT2* gene is responsible for the biosynthesis of the carbohydrate antigen Sd^a^. Both the enzyme and its cognate antigen display a restricted pattern of tissue expression and modulation in colorectal, gastric, and mammary cancers. In colorectal cancer, *B4GALNT2* is generally downregulated, but patients displaying higher expression survive longer. The sialyl Lewis^a^ and sialyl Lewis^x^ antigens are associated with malignancy. Their biosynthesis and that of Sd^a^ are mutually exclusive. Forced expression of *B4GALNT2* in colorectal cancer cell lines modulates the transcriptome towards lower malignancy, reducing stemness. These effects are independent of *B4GALNT2*-induced sLe^a^/sLe^x^ inhibition. Thus, *B4GALNT2* is a marker of better prognosis and a cancer-restraining enzyme in colorectal cancer, with a therapeutic potential.

**Abstract:**

Terminal carbohydrate structures are particularly relevant in oncology because they can serve as cancer markers and alter the phenotype of cancer cells. The Sd^a^ antigen and the sialyl Lewis^x^ and sialyl Lewis^a^ (sLe^x^ and sLe^a^) antigens are terminal structures whose biosynthesis is mutually exclusive. In this review, we describe the main features of the Sd^a^ antigen in cancer and its relationship with sLe^x/a^ antigens. Information was obtained from an extensive literature search and from The Cancer Genome Atlas (TCGA) public database. The Sd^a^ biosynthetic enzyme *B4GALNT2* undergoes downregulation in colorectal (CRC) and stomach cancer, while it is ectopically expressed by a minority of breast cancer (BRCA) patients. High expression of *B4GALNT2* is associated with better prognosis and a less malignant gene expression profile in CRC, while the opposite occurs in BRCA. The regulation of *B4GALNT2* expression in CRC is multifactorial, involving gene methylation and miRNA expression. Forced expression of *B4GALNT2* inhibited sLe^a^/sLe^x^ and reduced malignancy and stemness in cells constitutively expressing sLe^x/a^ antigens. However, consistent effects were observed upon *B4GALNT2* forced expression and in cells not expressing sLe^x/a^ antigens. Thus, *B4GALNT2* and the Sd^a^ antigen exert a tumor-restraining activity in CRC and probably other gastrointestinal cancers, independently of sLe^x/a^ antigens.

## 1. Introduction

The impact of glycosylation on cell behavior largely depends on the terminal portions of glycoconjugates. Examples of terminal structures include α2,3- and α2,6-linked sialic acids, polysialic acid, the AB0 blood group, and other fucosylated structures such as the sialyl Lewis^x^ (sLe^x^) and the sialyl Lewis^a^ (sLe^a^) antigens [1,2,3]. The Sd^a^ antigen is a terminal carbohydrate structure expressed on erythrocytes, in secretions [4], and in a few organs [5] by the vast majority of Caucasians. The Sd^a^ antigen was discovered independently by two groups in 1967 [6,7] and found to be inherited as a dominant character. Although the percentage of Sd^a^-negative individuals was found to be 4%, the percentage of individuals with “natural” anti Sd^a^ antibodies in serum was much lower. In urine and kidney, the major carrier of Sd^a^ is the urinary Tamm–Horsfall glycoprotein. In this review, we will focus on the role of Sd^a^ and of its biosynthetic enzyme *B4GALNT2* in cancer and its relationship with sLe^x^ and sLe^a^ antigens. However, it is worth mentioning that Sd^a^ plays a role in a wide variety of biological systems, including the attenuation of the phenotype in mouse models of muscular dystrophy [8,9] and bleeding disorders [10,11], in the equilibrium of the gut microbiota [12,13], in the prolificacy of sheep [14], in the xenotransplantation of pig organs [15], and in the inhibition of influenza virus infection [16,17].

## 2. The Sd^a^ Antigen: Structure and Biosynthesis

**Figure 1:**
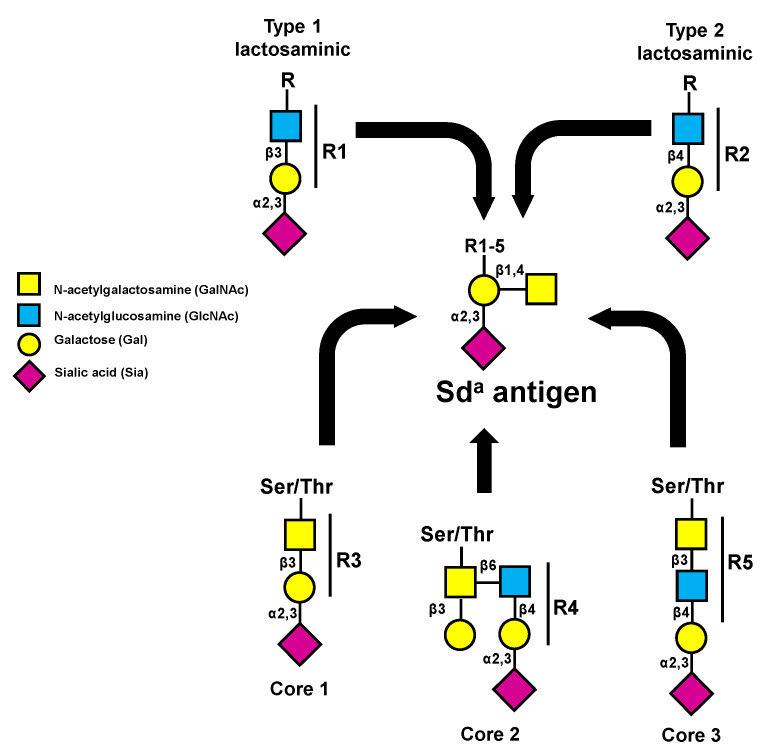
Structure of the different carbohydrate chains that can be terminated by the Sd^a^ antigen. These structures can be present on N- or O-linked chains. The addition of β1,4-linked GalNAc is always mediated by *B4GALNT2*.

The structure of the Sd^a^ antigen was elucidated in the 1980s and found to have a basic composition of a α2,3-sialylated galactose to which a β1,4 GalNAc is linked [18]. The underlying carbohydrate structure is variable, including type 1 (Galβ1,3GlcNAc) and type 2 (Galβ1,4GlcNAc) lactosaminic chains, as well as the core 1, core 2, and core 3 structures of O-linked chains (Figure 1) [19]. In addition, the glycolipid sialylparagloboside can be decorated by the Sd^a^ antigen [20]. 

**Figure 2:**
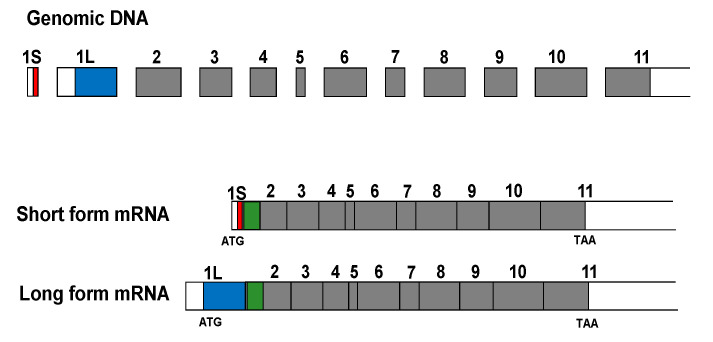
Structure of the genomic DNA and the two major *B4GALNT2* transcripts. Both the alternative exons 1S and 1L contain a translational start codon. Their coding portion is indicated in red and blue, respectively. Coding portions common to the two transcripts are in grey. The transmembrane portion is in green. Exons are drawn approximately to scale.

The addition of β1,4-linked GalNAc to α3-sialylated sugar chains is mediated by a single enzyme, product of the *B4GALNT2* gene. After the first identification of this enzymatic activity in Guinea pig kidney [21], the mouse *B4GALNT2* cDNA was cloned through a transient expression cloning approach [22]. Successively, the human cDNA was cloned from the colon cancer cell line Caco 2 by two different groups [23,24]. The human *B4GALNT2* gene maps on 17q21.33 and encompasses at least 12 coding exons. The presence of multiple transcripts diverging in their 5′- and 3′-UTR, some of which are up to 9000 nucleotides long, and the occurrence of at least two alternative first exons were documented [23,24]. The transcripts were mainly expressed in the colon and to a lower extent in ileum, stomach, and kidney. The alternative presence of the 253-base-pair-long exon 1L or of the 38-base-pair-long exon 1S leads to two transcript variants containing different translational start sites. Consequently, the human *B4GALNT2* gene can originate at least two different transmembrane peptides, diverging in their amino-terminal portion: a 566-amino acid long form and a 506-amino acid short form (Figure 2). 

Both isoforms display Golgi localization, although the long form is present also in post-Golgi vesicles and the plasma membrane [25]. The short form appears to be enzymatically more active than the long form [26] and is by far the dominant form in both normal and cancer colon [27]. The genetic bases of *B4GALNT2* deficiency linked to the Sd^a^-negative phenotype have been recently elucidated and found to be mainly associated with missense mutations in the C-terminal portion of the enzyme [28]. Although the most frequent mutation appears to be Cys466Arg, other mutant alleles, including one affecting mRNA splicing, have been described [28]. In spite of the high similarity between the Sd^a^ antigen and the sugar chain of ganglioside GM2, *B4GALNT2* is unable to synthesize the ganglioside GM2, which is instead the product of *B4GALNT1*. On the other hand, GM2 synthase B4GALNT1 is unable to synthesize the Sd^a^ antigen.

A role for DNA methylation in *B4GALNT2* gene regulation was suggested by the presence in the genomic regions upstream of exons 1L and 1S of the features of a CpG island. A paper showed that the *B4GALNT2* transcript was detectable by RT-PCR in some gastrointestinal cell lines and not in others, but only after 40 amplification cycles [29]. The promoter region of the cell lines not expressing *B4GALNT2* was found to be methylated, and gene expression was restored by treatment with the DNA methylation inhibitor 5’aza 2’-deoxycytidine [29]. Another study [30] found methylation of the *B4GALNT2* gene in the majority of gastric and colon cancer cell lines. A weak expression of the *B4GALNT2* transcript and of the Sd^a^ antigen was induced by treatment of cell lines with anti-DNA-methylation agents [30]. Recently, it has been shown that the transcription factors ETS1 and, to a lesser extent, SP1 are required for *B4GALNT2* transcription, although neither of the two is responsible for its differential expression in CRC [31].

## 3. Sd^a^/*B4GALNT2* in Development, Differentiation, and Cancer

### 3.1. Development and Differentiation

Ontogenic regulation of *B4GALNT2* was formerly suggested by the observation that the Guinea pig kidney enzyme showed a five-fold increase after birth [32], while in rat colon, the enzyme was nearly undetectable at birth, but its level rapidly raised after weaning [33]. Consistently, the Sd^a^ antigen was not detected in human fetal colonic mucins [34]. The dependence of Sd^a^/*B4GALNT2* on cell differentiation was unclear. In fact, while the enzyme activity of both Sd^a^ [35] and *B4GALNT2* [33] was higher in the poorly differentiated cells of the colonic crypt, *B4GALNT2* expression increased upon differentiation of the human colon cancer Caco2 cells [36]. 

### 3.2. Cancer

Cancer-dependent modulation of *B4GALNT2* was reported by numerous studies in CRC and by a few in gastric cancer. A possible involvement of *B4GALNT2* in the biology and clinic of breast cancer (BRCA) has only recently emerged. These studies involved the analysis of clinical specimens, as well as of cell lines. 

#### 3.2.1. Clinical Studies

Traditionally, the clinical impact of cancer-modulated glycosyltransferases was investigated in cohorts of patients specifically recruited for the study by research Institutions. Although this approach has provided invaluable contributions to the field, it is strongly limited by the relatively small number of specimens that can be collected and analyzed. This limitation has been circumvented by public databases reporting genomic, transcriptomic, epigenetic, and clinical data of hundreds or thousands of cancer patients. The most comprehensive of these databases is probably The Cancer Genome Atlas (TCGA) (https://portal.gdc.cancer.gov/, accessed on 14 August 2021).

##### Colon Cancer

The dramatic downregulation of *B4GALNT2* expression in colorectal cancer tissues was formerly reported in 1989 [37], successively confirmed [26] and found to be largely due to a reduced mRNA expression [38]. Moreover, the expression of the Sd^a^ antigen was reduced in colon cancer, compared with normal mucosa, paralleling the expression of the enzyme [39]. Recently, an association of *B4GALNT2* mRNA with ulcerative colitis, an inflammatory pre-neoplastic condition [40], has been reported. The *B4GALNT2* transcript was found to be more expressed in long-duration, compared with short-duration ulcerative colitis cases. 

**Figure 3:**
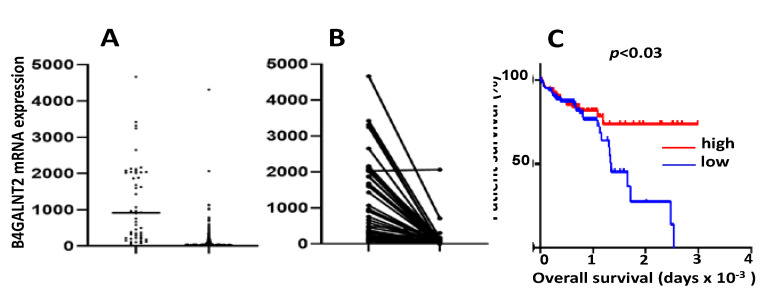
*B4GALNT2* mRNA in TCGA COADREAD (colon and rectal adenocarcinoma) cohort. (**A**): unpaired distribution of *B4GALNT2* expression in 49 normal samples and 626 cancer samples. Statistical analysis was conducted by the Mann–Whitney test. (**B**): *B4GALNT2* distribution in paired normal and tumor tissues of 49 patients. Statistical analysis was conducted by the Wilcoxon test. (**C**): Kaplan–Meier survival curves of patients belonging to the groups of high expressers (15th upper percentile, red) or no expressers (15th lower percentile, blue) of *B4GALNT2* mRNA, as deduced from the Oncolnc.org website.

TCGA data confirm the general downregulation of *B4GALNT2* in cancer tissues (Figure 3A) [41]. Although the vast majority of cancer samples lacked detectable levels of the transcript, several cancer cases displayed a level of expression comparable with that of many normal tissues. Paired comparison in 49 normal mucosa/cancer pairs (Figure 3B) revealed that only one sample displayed the same high expression in both the normal mucosa and the cancer. However, as previously observed in both European [26,37] and Japanese [38] studies, a small percentage of individuals expressing very low *B4GALNT2* levels in normal colon was present also in TCGA cohort. These individuals are likely Sd^a^-negative, although missense point mutations found to be responsible for several Sd^a^-negative cases [28] cannot be responsible for low-mRNA expression data.

According to these data, *B4GALNT2* mRNA level was nearly undetectable in the majority of cancer samples, but a remarkable number of cases retained a relatively high level of expression. These high-*B4GALNT2* expressers (HBE) often displayed a non-mucinous, microsatellite-stable phenotype and a better response to therapy [41]. However, the most remarkable clinical finding emerging from TCGA data was the close association of high *B4GALNT2* expression with long overall survival (Figure 3C). Patients stratified according to the *B4GALNT2* transcription level displayed different gene expression profiles. In fact, numerous genes showed different levels of expression in HBE, compared with low *B4GALNT2* expressers (LBE). In general, the gene expression profile of HBE is strongly oriented towards an attenuation of the neoplastic phenotype and the maintenance of functions associated with a normal epithelium, such as mucus secretion and solute transportation. Interestingly, the glycosylation machinery is also differentially expressed in HBE and LBE. In fact, several glycosyltransferases involved in the biosynthesis of O-linked chains, gangliosides, Lewis antigens, and galectins are more highly expressed in HBE, while ST6GAL sialyltransferases are more highly expressed in LBE [42].

**Figure 4:**
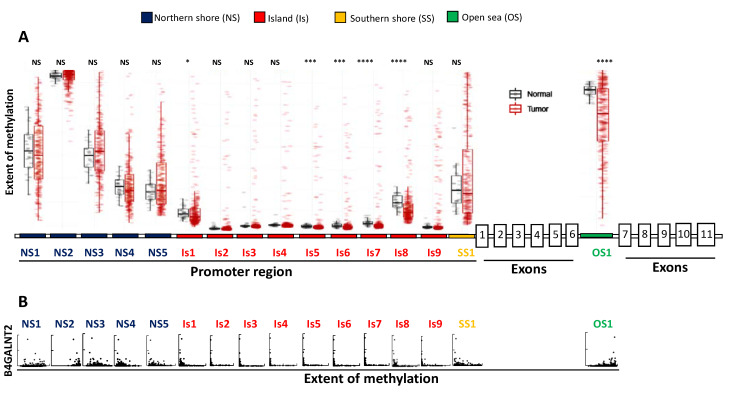
TCGA data on the methylation of the *B4GALNT2* gene in normal and CRC tissues. (**A**): extent of methylation in normal and CRC tissues of CpG positions in the NS (blue, five positions), CpG island (Is, red, nine positions), SS (yellow, one position), and OS (green, one position). The promoter region and the coding exons have not been drawn to scale. (**B**): *B4GALNT2* mRNA expression plotted vs. the extent of methylation in cancer tissues. * *p* < 0.05; *** *p* < 0.001; **** *p* < 0.0001.

The mechanisms regulating *B4GALNT2* expression in normal and cancer tissues are probably multifactorial and partially epigenetic. TCGA data allow a detailed analysis of the relationship between *B4GALNT2* expression and methylation of individual methylation sites located in the CpG island, as well as in its upstream and downstream flanking regions known as “northern shore (NS)” and “southern shore (SS)”, respectively [42] (Figure 4). In addition, the methylation of an “open sea (OS)” site located in the intron between exons 6 and 7 was also reported. In general, methylation of the island and of the northern and southern shore positions in colon cancer is closely associated with low or no *B4GALNT2* transcription. However, the methylation level in these positions is similar in the corresponding normal tissues (Figure 4A), ruling out the possibility that a differential methylation of these sites can be responsible for the downregulation of *B4GALNT2* in cancer. The most important regulatory role appears to be played by the OS1 position, whose methylation is lower in cancer. As a common condition, all HBE displayed high methylation of the NS2 and OS1 sites and lack of methylation of all other positions (Figure 4B). However, not all cases sharing this condition are HBE, suggesting this is a necessary but not sufficient condition. 

TCGA also provides information on the level of expression of numerous miRNA. Bioinformatics analysis has revealed that some miRNAs are potentially able to target *B4GALNT2* [42]. TCGA data showed a clear tendency towards an inverse relationship between some of these miRNAs and *B4GALNT2* in colon cancer tissues. However, hsa-miR-204-5p, that targets a sequence located in the 3’-UTR, about 120 bp downstream of the translational stop codon, displayed the best inverse relationship with *B4GALNT2* expression. In fact, all HBE displayed no hsa-miR-204-5p expression, while all high-hsa-miR-204-5p expressers displayed no *B4GALNT2* expression. However, the existence of many hsa-miR-204-5p non-expressers failing to express *B4GALNT2* revealed that the lack of hsa-miR-204-5p is also a necessary but not sufficient condition for high levels of *B4GALNT2*.

In conclusion, the regulation of *B4GALNT2* in normal and cancer colon is complex and multifactorial. The differential expression of specific transcription factors has not yet been investigated but is likely to play a major role in *B4GALNT2* regulation. 

##### Gastric Cancer

Using monoclonal antibodies specific for the Sd^a^ antigen and/or for the ganglioside GM2, it was found that the two structures displayed opposite regulation in normal gastric mucosa and gastric cancer; the former was downregulated, while the latter was upregulated in cancer [43]. The downregulation of *B4GALNT2* in gastric cancer was documented successively [38]. A detailed structural analysis in sera identified eight carbohydrate structures expressing the Sd^a^ antigen on the various underlying structures depicted in Figure 1 [44]. Interestingly, in sera of healthy people the Sd^a^ antigen was expressed only on O-linked Core 1 structures. On the other hand, in sera of a few gastric (and a few pancreatic) cancer patients, these Sd^a^ structures were found to be carried by other underlying chains [44]. The importance of these markers in cancer management requires further investigation.

**Figure 5:**
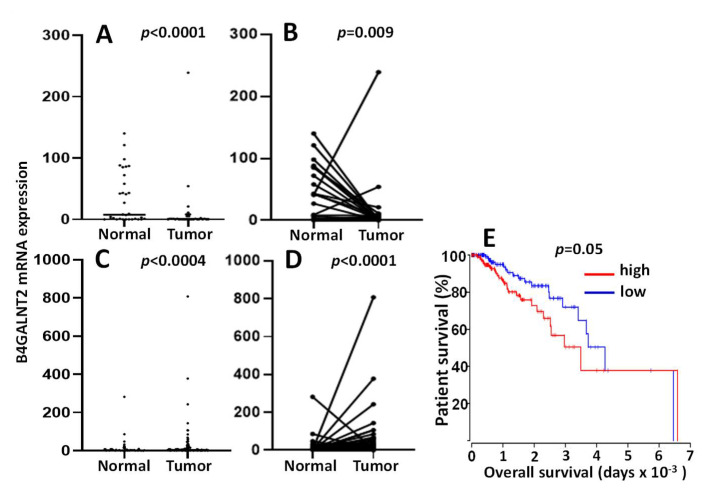
*B4GALNT2* mRNA in stomach adenocarcinoma (STAD) and breast cancer (BRCA) TCGA cohorts. In (**A**) and (**C**) plots, the unpaired distribution of *B4GALNT2* expression in normal tissue and cancer samples of stomach (**A**) and breast (**C**) cancer patients is reported. In (**B**) and (**D**) graphs, *B4GALNT2* distribution in paired normal and tumor tissues of stomach (**B**) and breast (**D**) cancer patients is reported. Stomach: 35 normal and 415 tumor samples; breast: 107 normal and 1100 cancer patients. Statistical analysis conducted by the Mann–Whitney test in **A** and **C** and by the Wilcoxon test in (**B**) and (**D**). (**E**): Kaplan–Meier survival curves of patients belonging to the groups of high expressers (15th upper percentile, red) or no expressers (15th lower percentile, blue) of mRNA *B4GALNT2* as deduced from the Oncolnc.org website.

According to TCGA data, *B4GALNT2* is expressed in the gastric mucosa by only a few cases, while it is virtually not expressed in gastric cancer, with few exceptions (Figure 5A,B). Overall, the level of *B4GALNT2* expression in the gastric mucosa is about 50-fold lower than in the colon. In normal stomach, *B4GALNT2* activity and Sd^a^ antigen expression were found to be associated with chief cells, the stomach cells releasing pepsinogen [43]. Methylation of the promoter was shown to be relevant for *B4GALNT2* expression by the two previously mentioned studies [29,30] as well as in gastric cancer cell lines. However, the low level of expression in gastric cancer tissues did not allow investigating the relationship between *B4GALNT2* expression and methylation or clinical features using TCGA data.

##### Breast Cancer

Little is known about *B4GALNT2* in BRCA. A recent study reported a systemic upregulation of *B4GALNT2* in an experimental model of breast cancer [45]. Consistently, TCGA data analysis showed that in normal breast tissues as well as in the majority of BRCA tissues, the level of *B4GALNT2* mRNA expression was nearly undetectable (Figure 5C,D), although a minority of the cancer cases displayed a level of activity similar with that of colon. Considering the virtual absence of *B4GALNT2* in normal breast tissues, it seems appropriate to define the *B4GALNT2* expression by a minority of BRCA cases as ectopic. Notably, these high-*B4GALNT2* expressers displayed a significantly shorter overall survival (Figure 5E). No obvious relationship was evident with clinical parameters, including receptor (estrogens, progesterone, or HER2) status. However, genes displaying over- or under-expression in the HBE cohorts provided a molecular signature strongly oriented towards malignancy (Table 1). This view was confirmed by a recent study showing growth promotion induced by *B4GALNT2* in triple-negative breast cancer cell lines [46].

## 4. Sialyl Lewis Antigens 

sLe^x^ and sLe^a^ are well known cancer-associated fucosylated carbohydrate structures [47]. Although they are normally expressed by a variety of tissues, including leukocytes, their ectopic expression by several cancers is associated with malignancy [48]. The sLe^a^ tetrasaccharide is the epitope of the CA19.9 antigen, widely used in clinical practice [49,50,51]. 

### 4.1. Structure and Biosynthesis in Normal and Cancer Colon

The terminal steps of the biosynthesis of the sLe^x^ and sLe^a^ antigens are mediated by α2,3 sialyltransferases and successively by α1,3/4 fucosyltransferases [52]. While type 1 chains can be α2,3 sialylated only by ST3GAL3, type 2 chains can be α2,3 sialylated by ST3GAL3, ST3GAL4, and ST3GAL6. The successive addition of fucose in α1,4 linkage to type 1 chains is catalyzed only by FUT3, while that in α1,3 linkage to type 2 chains in colon cancer is mediated mainly, if not exclusively, by FUT6 [53]. The usage of these different enzymes is strongly cell-type specific. 

**Figure 6:**
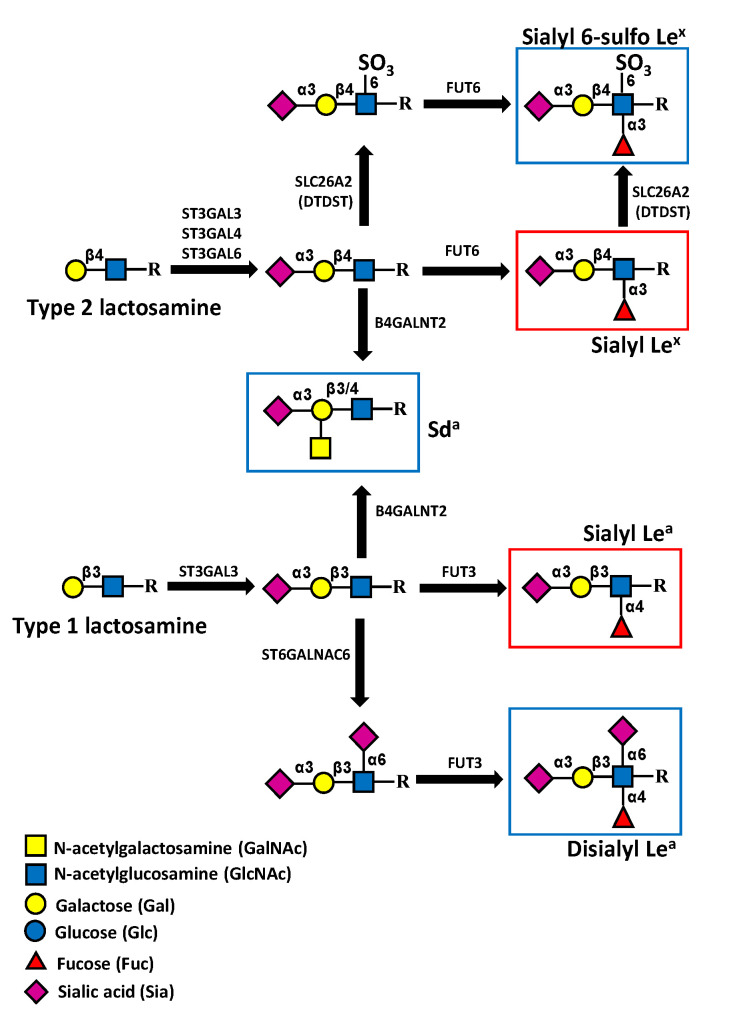
Sialyl Lewis-related antigens and Sd^a^ antigen in colonic tissues. In normal colon, the expression of sialyl 6-sulfo Le^x^ antigen (upper) predominates over that of sLe^x^. However, the reduced expression of enzymes responsible for its biosynthesis, such as *SLC26A2* (formerly DTDST) in colon cancer, leads to the overexpression of sLe^x^. The biosynthesis of the di-sialyl Le^a^ antigen (lower), which predominates in normal colon, proceeds from α2,6 sialylation, catalyzed by *ST6GALNAC6*, followed by α1,4-fucosylation mediated by FUT3. The downregulation of *ST6GALNAC6* in colon cancer leads to sLe^a^ overexpression. The addition of β1,4-linked GalNAc mediated by *B4GALNT2* leads to the expression of the Sd^a^, inhibiting that of sLe^x^ and sLe^a^. Cancer-associated structures are boxed in red, while structures associated with normal colon are boxed in blue.

In CRC, the aberrant expression of sLe^x/a^ antigens appears to be particularly relevant [54,55,56,57,58,59,60] (recently reviewed in [61]). However, the molecular bases of their overexpression in this malignancy are unclear. Although several studies have shown that some of the above-mentioned α2,3 sialyltransferases [62,63,64,65,66,67] and α1,3/4 fucosyltransferases [53,68,69,70] have the ability to regulate sLe^x/a^ biosynthesis in experimental systems, the overexpression of these antigens in CRC is not due to the increased expression of their cognate sialyl- and/or fucosyltransferases [53,71]. In fact, the level of expression of sLe^x/a^ antigens does not reflect the level of expression of these terminal glycosyltransferases in normal and CRC tissues. In normal colon, sLe^x^ is expressed by only a few specimens, and the level of antigen expression is much lower than that in cancer samples [53], but the expression level of *FUT6* is quite similar in normal and cancer colon tissues [53,71]. Glycosyltransferases synthesizing subterminal carbohydrate structures can play crucial roles. In particular, the GlcNAc transferases *B3GNT5* and *B3GNT7* appear to play opposite roles. The first one, which is involved in the biosynthesis of the underlying type 1 and type 2 chains, promotes sLe^x/a^ overexpression [72,73], whereas the second one, which extends sulfated polylactosaminic chains, inhibits sLe^x/a^ biosynthesis [74]. Overexpression of sialidases, in particular NEU4, which acts preferentially on mucins and is downregulated in CRC, has been shown to play a role in keeping low levels of sLe^x/a^ in normal colon and, consequently, higher levels in CRC [75]. Among the mechanisms that have been claimed to be responsible for the upregulation of sLe^x/a^ in CRC, the biosynthesis in normal colonic tissues of carbohydrate structures whose expression prevents that of sLe^x/a^ antigens is particularly relevant. An example is provided by the antigen sialyl 6-sulfo Le^x^, highly expressed in normal colon, which is replaced in CRC by sLe^x^, due to reduced sulfation in the latter (Figure 6) [76]. The process is mainly regulated by the epigenetic silencing of the sulfate transporter DTDST, the product of the *SLC26A2* gene [77]. Another example is provided by the alternative biosynthesis of di-sialyl Lewis^a^ or sLe^a^ antigens in normal mucosa and CRC, respectively [78]. The former, mainly expressed by normal mucosa, is synthesized by the sequential action of sialyltransferase *ST6GALNAC6* and fucosyltransferase FUT3 (Figure 6). However, when *ST6GALNAC6* is downregulated, as occurs in CRC, only the sLe^a^ antigen is synthesized. It has been recently reported that biglycan (encoded by the *BGN* gene) is responsible for the epigenetic silencing of the *SLC26A2* and *ST6GALNA6* genes in CRC through an inflammatory pathway described below [79,80]. A third example of biosynthetic competition between different antigens is provided by sLe^x/a^ and Sd^a^. Both derive from a common precursor, i.e., an α2,3-sialylated type 1 or type 2 chain. The addition of β1,4-linked GalNAc to Gal hinders the addition of fucose to GlcNAc and vice versa [26,81]. In fact, a structural analysis of mucins from normal colon specimens revealed a large preponderance of oligosaccharides frequently terminated by the Sd^a^ epitope [82], while sLe^x^ structures were minor components. Interestingly, no structures carrying both the Sd^a^ and the sLe^x^ determinants were detected. We have previously proposed that the right question to answer is not “why is sLe^x^ high in colon cancer?” but rather: “why is sLe^x^ low in normal colon?” The reduced *B4GALNT2* expression in CRC is part of the answer. In fact, a significant relationship in normal colonic mucosa between the sLe^x^ level and the *FUT6*/*B4GALNT2* ratio has been reported [27], supporting the notion that in normal colon, sLe^x^ is poorly expressed because of a high level of *B4GALNT2*. 

### 4.2. Role in Malignancy

sLe^x/a^ structures act as ligands for cell adhesion molecules of the selectin family, playing a fundamental role in leukocyte extravasation [83]. However, when overexpressed in cancer cells, they contribute to metastasis formation [84], particularly because they allow the interaction of circulating cancer cells with selectins expressed on endothelial cells [85,86,87,88,89]. This notion has been clearly demonstrated in vivo by showing that a CRC cell line injected subcutaneously in immunodeficient mice formed a much lower number of spontaneous lung metastases in E- and P-selectin-deficient mice [90]. In addition, E- and P-selectins were found to be crucial for peritoneal metastasization of pancreatic cancer cells [91] and bone metastasis of breast cancer cells [92]. Recently, it has been shown that sLe antigens are involved in bone metastasis formation by mediating adhesion to the bone niche [93]. Inflammation is a crucial feature associated with cancer, and cancer frequently develops on pre-existing inflammatory conditions. In inflammatory bowel diseases, sLe^x/a^ antigens expressed by mucosal cells on the CD44v6 molecule sustain inflammation by acting as ligands for transmigrating neutrophils [94]. Siglecs are sialic acid-binding molecules with a general anti-inflammatory and immunosuppressive activity [95]. The above-mentioned antigens sialyl 6-sulfo Le^x^ and di-sialyl Lewis^a^, expressed by normal mucosa, are ligands for Siglec-7. Consequently, their downregulation in CRC contributes to the cancer-associated inflammatory status [79,80]. Biglycan, overexpressed in CRC, is a good ligand for Toll-like receptor 4 (TLR4) [73,74]. This leads to the activation in CRC of the TLR4/NFkB inflammatory pathway, resulting in the previously mentioned epigenetic silencing of the *SLC26A2* and *ST6GALNA6* genes, responsible for sialyl 6-sulfo Le^x^ and di-sialyl Lewis^a^ biosynthesis [79,80].

Apart from the increased adhesion to selectin-expressing vessels, several studies indicate that sLe^x/a^ structures increase motility, proliferation, and malignancy through selectin-independent mechanisms [87,88,96,97,98]. The colon cancer cell lines SW480 and SW620, which were derived from a primary colon cancer and a lymph node metastasis of the same patient, respectively, provide a good model of cells with different malignancy but a very similar genetic background. Forced expression of *FUT6* in these cells resulted in profound changes of gene expression towards increased malignancy [97]. However, some genes were modulated only in one of the two cell lines, while others in both, despite the close relationship between the two cell lines. For example, transcription of a group of genes strongly involved in DNA replication, including those encoding telomerase (*TERT),* thymidylate synthase (*TYMS*), and DNA polymerase ε4 *(POLE4*), was stimulated by *FUT6* in SW620 but not in SW480 [97]. Furthermore, the impact of *FUT6*/sLe^x^ on the phenotype of the two cell lines was different. In fact, the clonogenic ability in soft agar and the capacity to heal a wound were increased only in SW620 but not in SW480 [97].

## 5. Phenotypic Effects of *B4GALNT2* on Cancer Phenotype

In vitro experiments have shown that forced expression of *B4GALNT2* in colon and gastric cancer cell lines strongly reduced sLe^x/a^ expression [26,81]. In addition, *B4GALNT2*-expressing cells showed reduced metastatic ability [81,99]. More recently, we have shown that in the colon cancer cell line LS174T that constitutively expresses sLe^x/a^, *B4GALNT2* reduced stemness-associated features, in particular the ability to grow in poor adherence. Notably, *B4GALNT2* strongly modulated the transcriptional activity towards an attenuation of the neoplastic phenotype in this cell line [41]. It has long been thought that the tumor-restraining activity exerted by *B4GALNT2*/Sd^a^ was dependent on sLe^x/a^ inhibition, rather than on de novo expression of Sd^a^. However, this view has been challenged by the observation that in SW480 and SW620 cells (lacking sLe^x^ if not transfected with *FUT6*), forced expression of *B4GALNT2* [97] downregulated all malignancy-associated phenotypes (including cell proliferation, growth in poor adherence, wound healing ability, and stemness marker expression) in SW620, but only those associated with stemness in SW480 [97]. Thus, attenuation of the stemness-associated malignant phenotype by *B4GALNT2*/Sd^a^ appears to be a common feature in CRC cells and is independent of sLe^x^ inhibition. 

## 6. Discussion

In this review, we have described the mutual relationship of Sd^a^ and sLe^x/a^ antigens, showing how the terminal portions of glycoconjugates can play profound but sometimes opposite effects on cancer biology. The data summarized in this review reinforce or challenge established paradigms. First, the ectopic expression of sLe^x/a^ antigens in CRC would be due, together with other factors, to the downregulation of *B4GALNT2* rather than to the upregulation of the sLe^x/a^ biosynthetic machinery (graphical abstract). Second, the Sd^a^ synthase *B4GALNT2* is associated with better prognosis in CRC but with worse prognosis in BRCA. Moreover, the gene expression profile of HBE in CRC and BRCA is opposite. This supports the notion that the impact of a glycosyltransferase on cancer is strongly tissue-dependent. Third, Sd^a^/*B4GALNT2* inhibits numerous properties of malignancy in CRC cells, in particular those associated with stemness. However, this effect is not dependent on sLe^x/a^ inhibition, as previously thought. It remains to be established whether cell lines from other organs of the gastrointestinal tract, as well as from BRCA, display this effect. Fourth, the regulation of a glycosyltransferases can be very complex and multifactorial. *B4GALNT2* is regulated by DNA methylation, although the crucial sites appear to be located outside the canonical CpG island. miRNAs also play a role. However, appropriate patterns of methylation and miRNA expression appear to be a necessary but not sufficient condition for high expression. It is possible to hypothesize that a differential expression of key transcription factors plays a pivotal role during the complete or partial shut-down of *B4GALNT2* transcription associated with CRC transformation. The identification of these transcription factors deserves investigation. Fifth, glycosyltransferases impact the gene expression profile and the phenotype of cell lines. Consistent with clinical data, overexpression of *B4GALNT2* attenuates malignancy, while overexpression of *FUT6*/sLe^x^ exacerbates malignancy, although in a strongly cell-type specific manner. While the use of sLe antigens as cancer markers has been debated since the 1980s and is already applied in the clinic (CA19.9), the use of Sd^a^ and/or *B4GALNT2* as prognostic markers would be novel. The detection of the Sd^a^ antigen is limited by the lack of a commercially available antigen and by its poor detection on formalin-fixed/paraffin-embedded tissues (F. Dall’Olio, unpublished observation). On the contrary, the precise quantification in clinical samples of *B4GALNT2* mRNA by RNA-seq techniques at a cost compatible with clinical routine, would be a realistic perspective. 

## 7. Conclusions

In conclusion, *B4GALNT2* and its cognate carbohydrate antigen Sd^a^ play a relevant role in CRC, not only for their association with better prognosis, but also because their expression is causally related to reduced malignancy in CRC experimental systems. For these reasons, their use as clinical markers deserves consideration. The reduced malignancy induced by the forced expression of *B4GALNT2* in cancer cells by viral vectors [99] also suggests its potential as a therapeutic agent.

## Figures and Tables

**Table 1:** Cancer-relevant genes up- or downregulated in HBE expressers.

Gene Symbol	Ratio	Function	PMID	
*KRT20*	494	Cytokeratin 20, associated with worse prognosis	22493626	
*CEACAM6*	11	Member of the CEA family. Associated with worse prognosis	24186057	
*CRISP3*	10	High expression correlates with malignancy	30609035	
*ABCC2*	10	Multidrug resistance-associated protein 2	26499806	
*LRRC31*	9	Inhibitor of DNA repair	33005030	
*MUC21*	8.5	Associated with incohesive growth of lung cancer	31301084	
*CEACAM5*	8	Member of the CEA family. Driver of breast cancer metastasis	29736411	
*TRIM72*	8	Lower expression predicts recurrence in colon cancer	30852740	
*PNMT*	7.8	Co-amplified with ERBB2	12727839	
*CXCL17*	7	Promotes proliferation and invasion of breast cancer cells	28943434	
*AMER3*	−8.7	Enhances β-catenin signaling in CRC	24251807	
*KCNC1*	−8.8	Its inhibition is associated with poor survival in seminoma	34105734	
*INSM1*	−9.3	In SCLC, low expression associated with better prognosis	32118626	
*SLIT1*	−9	Suppresses breast cancer growth	18829537	
*RUNDC3A*	−10.6	High expression correlates with shorter survival in rectal cancer	29050227	
*HDC*	−13	Increased expression associated with better survival	31748740	
*ECEL1*	−14	Associated with good prognosis in neuroblastoma	12632073	
*CHRNA9*	−19	High expression associated with poor survival in BRCA	20953833	
*PCSK1*	−23	Reduces growth of breast cancer cells	11241161	
*NELL1*	−27	Down-regulated in kidney cancer	25726761	

Top 20 genes showing significantly (*p* ≤ 0.05 by Student’s t test) higher (10 genes) or lower (10 genes) expression in the 15% upper percentile of *B4GALNT2* expression. The red or green color indicates that the change has a putatively cancer-promoting (red) or cancer-restraining (green) effect, according to the literature search. The “PubMed Identification Number” (PMID) of the most relevant paper is indicated.

## Abbreviations

BRCA	breast cancer
COADREAD	colon adenocarcinoma rectal adenocarcinoma
CRC	colorectal cancer
DTDST	diastrophic dysplasia sulfate transporter
HBE	high-*B4GALNT2* expressers
LBE	low-*B4GALNT2* expressers
NS	northern shore
OS	open sea
RT-PCR	reverse transcriptase-polymerase chain reaction
sLe^a^	sialyl Lewis^a^
sLe^x^	sialyl Lewis^x^
SCLC	small cell lung cancer
SS	southern shore
STAD	stomach adenocarcinoma
TCGA	The Cancer Genome Atlas
TLR4	Toll-like receptor 4

## References

[1] Dall’Olio F., Malagolini N., Trinchera M., Chiricolo M. (2012). Mechanisms of cancer-associated glycosylation changes. Front. Biosci..

[2] Dall’Olio F., Malagolini N., Trinchera M., Chiricolo M. (2014). Sialosignaling: Sialyltransferases as engines of self-fueling loops in cancer progression. Biochim. Biophys. Acta.

[3] Pinho S.S., Reis C.A. (2015). Glycosylation in cancer: Mechanisms and clinical implications. Nat. Rev. Cancer.

[4] Morton J.A., Pickles M.M., Terry A.M. (1970). The Sd^a^ blood group antigen in tissues and body fluids. Vox Sang..

[5] Morton J.A., Pickles M.M., Vanhegan R.I. (1988). The Sd^a^ antigen in the human kidney and colon. Immunol. Investig..

[6] Renton P.H., Howell P., Ikin E.W., Giles C.M., Goldsmith K.L. (1967). Anti Sd^a^: A new blood group antibody. Vox Sang..

[7] Macvie S.I., Morton J.A., Pickles M.M. (1967). The reactions and inheritance of a new blood group antigen. Vox Sang..

[8] Cramer M.L., Xu R., Martin P.T. (2019). Soluble Heparin Binding EGF-like Growth Factor (HB-EGF) is a regulator of GALGT2 expression and GALGT2-dependent muscle and neuromuscular phenotypes. Mol. Cell Biol..

[9] Xu R., Singhal N., Serinagaoglu Y., Chandrasekharan K., Joshi M., Bauer J.A., Janssen P.M., Martin P.T. (2015). Deletion of Galgt2 (B4Galnt2) Reduces Muscle Growth in Response to Acute Injury and Increases Muscle Inflammation and Pathology in Dystrophin-Deficient Mice. Am. J. Pathol..

[10] Vallier M., Abou C.M., Hindersin L., Linnenbrink M., Traulsen A., Baines J.F. (2017). Evaluating the maintenance of disease-associated variation at the blood group-related gene B4galnt2 in house mice. BMC Evol. Biol..

[11] Linnenbrink M., Johnsen J.M., Montero I., Brzezinski C.R., Harr B., Baines J.F. (2011). Long-term balancing selection at the blood group-related gene B4galnt2 in the genus Mus (Rodentia; Muridae). Mol. Biol. Evol..

[12] Staubach F., Kunzel S., Baines A.C., Yee A., McGee B.M., Backhed F., Baines J.F., Johnsen J.M. (2012). Expression of the blood-group-related glycosyltransferase B4galnt2 influences the intestinal microbiota in mice. ISME J..

[13] Galeev A., Suwandi A., Cepic A., Basu M., Baines J.F., Grassl G.A. (2021). The role of the blood group-related glycosyltransferases FUT2 and B4GALNT2 in susceptibility to infectious disease. Int. J. Med. Microbiol..

[14] Ben J.S., Ruesche J., Sarry J., Woloszyn F., Lassoued N., Fabre S. (2018). The high prolificacy of D’man sheep is associated with the segregation of the FecL(L) mutation in the B4GALNT2 gene. Reprod. Domest. Anim..

[15] Byrne G., Ahmad-Villiers S., Du Z., McGregor C. (2018). B4GALNT2 and xenotransplantation: A newly appreciated xenogeneic antigen. Xenotransplantation.

[16] Heaton B.E., Kennedy E.M., Dumm R.E., Harding A.T., Sacco M.T., Sachs D., Heaton N.S. (2017). A CRISPR Activation Screen Identifies a Pan-avian Influenza Virus Inhibitory Host Factor. Cell Rep..

[17] Wong H.H., Fung K., Nicholls J.M. (2019). MDCK-B4GalNT2 cells disclose a a2,3-sialic acid requirement for the 2009 pandemic H1N1 A/California/04/2009 and NA aid entry of A/WSN/33. Emerg. Microbes. Infect..

[18] Donald A.S., Yates A.D., Soh C.P., Morgan W.T., Watkins W.M. (1983). A blood group Sd^a^-active pentasaccharide isolated from Tamm-Horsfall urinary glycoprotein. Biochem. Biophys. Res. Commun..

[19] Dall’Olio F., Malagolini N., Chiricolo M., Trinchera M., Harduin-Lepers A. (2014). The expanding roles of the Sd^a^/Cad carbohydrate antigen and its cognate glycosyltransferase B4GALNT2. Biochim. Biophys. Acta.

[20] Blanchard D., Piller F., Gillard B., Marcus D., Cartron J.P. (1985). Identification of a novel ganglioside on erythrocytes with blood group Cad specificity. J. Biol. Chem..

[21] Serafini-Cessi F., Dall’Olio F. (1983). Guinea-pig kidney b-N-acetylgalactosaminyltransferase towards Tamm- Horsfall glycoprotein. Requirement of sialic acid in the acceptor for transferase activity. Biochem. J..

[22] Smith P.L., Lowe J.B. (1994). Molecular cloning of a murine N-acetylgalactosamine transferase cDNA that determines expression of the T lymphocyte-specific CT oligosaccharide differentiation antigen. J. Biol. Chem..

[23] Lo Presti L., Cabuy E., Chiricolo M., Dall’Olio F. (2003). Molecular Cloning of the Human b1,4 N-Acetylgalactosaminyltransferase Responsible for the Biosynthesis of the Sd^a^ Histo-Blood Group Antigen: The Sequence Predicts a Very Long Cytoplasmic Domain. J. Biochem..

[24] Montiel M.D., Krzewinski-Recchi M.A., Delannoy P., Harduin-Lepers A. (2003). Molecular cloning, gene organization and expression of the human UDP-GalNAc:Neu5Aca2-3Galb-R b1,4-N-acetylgalactosaminyltransferase responsible for the biosynthesis of the blood group Sda/Cad antigen: Evidence for an unusual extended cytoplasmic domain. Biochem. J..

[25] Groux-Degroote S., Schulz C., Cogez V., Noel M., Portier L., Vicogne D., Solorzano C., Dall’Olio F., Steenackers A., Mortuaire M. (2018). The extended cytoplasmic tail of the human B4GALNT2 is critical for its Golgi targeting and post-Golgi sorting. FEBS J..

[26] Malagolini N., Santini D., Chiricolo M., Dall’Olio F. (2007). Biosynthesis and expression of the Sd^a^ and sialyl Lewis x antigens in normal and cancer colon. Glycobiology.

[27] Groux-Degroote S., Wavelet C., Krzewinski-Recchi M.A., Portier L., Mortuaire M., Mihalache A., Trinchera M., Delannoy P., Malagolini N., Chiricolo M. (2014). *B4GALNT2* gene expression controls the biosynthesis of Sd^a^ and sialyl Lewis X antigens in healthy and cancer human gastrointestinal tract. Int. J. Biochem. Cell Biol..

[28] Stenfelt L., Hellberg A., Moller M., Thornton N., Larson G., Olsson M.L. (2019). Missense mutations in the C-terminal portion of the *B4GALNT2*-encoded glycosyltransferase underlying the Sd^a^ phenotype. Biochem. Biophys. Rep..

[29] Wang H.R., Hsieh C.Y., Twu Y.C., Yu L.C. (2008). Expression of the human Sd^a^ b-1,4-N-acetylgalactosaminyltransferase II gene is dependent on the promoter methylation status. Glycobiology.

[30] Kawamura Y.I., Toyota M., Kawashima R., Hagiwara T., Suzuki H., Imai K., Shinomura Y., Tokino T., Kannagi R., Dohi T. (2008). DNA hypermethylation contributes to incomplete synthesis of carbohydrate determinants in gastrointestinal cancer. Gastroenterology.

[31] Wavelet-Vermuse C., Groux-Degroote S., Vicogne D., Cogez V., Venturi G., Trinchera M., Brysbaert G., Krzewinski-Recchi M.A., Bachir E.H., Schulz C. (2021). Analysis of the proximal promoter of the human colon-specific *B4GALNT2* (Sd^a^ synthase) gene: *B4GALNT2* is transcriptionally regulated by ETS1. Biochim. Biophys. Acta Gene Regul. Mech..

[32] Dall’Olio F., Malagolini N., Serafini-Cessi F. (1987). Tissue distribution and age-dependent expression of b-4-N- acetylgalactosaminyl-transferase in guinea-pig. Biosci. Rep..

[33] Dall’Olio F., Malagolini N., Di Stefano G., Ciambella M., Serafini-Cessi F. (1990). Postnatal development of rat colon epithelial cells is associated with changes in the expression of the b 1,4-N-acetylgalactosaminyltransferase involved in the synthesis of Sda antigen and of a 2,6-sialyltransferase activity towards N-acetyllactosamine. Biochem. J..

[34] Robbe-Masselot C., Maes E., Rousset M., Michalski J.C., Capon C. (2009). Glycosylation of human fetal mucins: A similar repertoire of O-glycans along the intestinal tract. Glycoconj. J..

[35] Lefrancois L. (1987). Carbohydrate differentiation antigens of murine T cells: Expression on intestinal lymphocytes and intestinal epithelium. J. Immunol..

[36] Malagolini N., Dall’Olio F., Serafini-Cessi F. (1991). UDP-GalNAc:NeuAc a 2,3Gal b-R (GalNAc to Gal) b 1,4-N- acetylgalactosaminyltransferase responsible for the Sda specificity in human colon carcinoma CaCo-2 cell line. Biochem. Biophys. Res. Commun..

[37] Malagolini N., Dall’Olio F., Di Stefano G., Minni F., Marrano D., Serafini-Cessi F. (1989). Expression of UDP-GalNAc:NeuAc a2,3Gal b-R beta 1,4(GalNAc to Gal) N-acetylgalactosaminyltransferase involved in the synthesis of Sd^a^ antigen in human large intestine and colorectal carcinomas. Cancer Res..

[38] Dohi T., Yuyama Y., Natori Y., Smith P.L., Lowe J.B., Oshima M. (1996). Detection of N-acetylgalactosaminyltransferase mRNA which determines expression of Sda blood group carbohydrate structure in human gastrointestinal mucosa and cancer. Int. J. Cancer.

[39] Robbe-Masselot C., Herrmann A., Maes E., Carlstedt I., Michalski J.C., Capon C. (2009). Expression of a core 3 disialyl-Le^x^ hexasaccharide in human colorectal cancers: A potential marker of malignant transformation in colon. J. Proteome. Res..

[40] Low E.N.D., Mokhtar N.M., Wong Z., Raja Ali R.A. (2019). Colonic Mucosal Transcriptomic Changes in Patients with Long-Duration Ulcerative Colitis Revealed Colitis-Associated Cancer Pathways. J. Crohns. Colitis..

[41] Pucci M., Gomes Ferreira I., Orlandani M., Malagolini N., Ferracin M., Dall’Olio F. (2020). High Expression of the Sd^a^ Synthase B4GALNT2 Associates with Good Prognosis and Attenuates Stemness in Colon Cancer. Cells.

[42] Pucci M., Malagolini N., Dall’Olio F. (2021). Glycosyltransferase B4GALNT2 as a Predictor of Good Prognosis in Colon Cancer: Lessons from Databases. Int. J. Mol. Sci..

[43] Dohi T., Ohta S., Hanai N., Yamaguchi K., Oshima M. (1990). Sialylpentaosylceramide detected with anti-GM2 monoclonal antibody. Structural characterization and complementary expression with GM2 in gastric cancer and normal gastric mucosa. J. Biol. Chem..

[44] Tanaka-Okamoto M., Hanzawa K., Mukai M., Takahashi H., Ohue M., Miyamoto Y. (2018). Identification of internally sialylated carbohydrate tumor marker candidates, including Sda/CAD antigens, by focused glycomic analyses utilizing the substrate specificity of neuraminidase. Glycobiology.

[45] Qusa M.H., Abdelwahed K.S., Siddique A.B., El Sayed K.A. (2021). Comparative Gene Signature of (-)-Oleocanthal Formulation Treatments in Heterogeneous Triple Negative Breast Tumor Models: Oncological Therapeutic Target Insights. Nutrients.

[46] Yu P., Zhu L., Cui K., Du Y., Zhang C., Ma W., Guo J. (2021). *B4GALNT2* Gene Promotes Proliferation, and Invasiveness and Migration Abilities of Model Triple Negative Breast Cancer (TNBC) Cells by Interacting With HLA-B Protein. Front Oncol..

[47] Holst S., Wuhrer M., Rombouts Y. (2015). Glycosylation characteristics of colorectal cancer. Adv. Cancer Res..

[48] Blanas A., Sahasrabudhe N.M., Rodriguez E., van Kooyk Y., van Vliet S.J. (2018). Fucosylated Antigens in Cancer: An Alliance toward Tumor Progression, Metastasis, and Resistance to Chemotherapy. Front Oncol..

[49] Aronica A., Avagliano L., Caretti A., Tosi D., Bulfamante G.P., Trinchera M. (2017). Unexpected distribution of CA19.9 and other type 1 chain Lewis antigens in normal and cancer tissues of colon and pancreas: Importance of the detection method and role of glycosyltransferase regulation. Biochim. Biophys. Acta Gen. Subj..

[50] Mare L., Caretti A., Albertini R., Trinchera M. (2013). CA19.9 antigen circulating in the serum of colon cancer patients: Where is it from?. Int. J. Biochem. Cell Biol..

[51] Trinchera M., Aronica A., Dall’Olio F. (2017). Selectin Ligands Sialyl-Lewis a and Sialyl-Lewis x in Gastrointestinal Cancers. Biology.

[52] Ferreira J.A., Magalhaes A., Gomes J., Peixoto A., Gaiteiro C., Fernandes E., Santos L.L., Reis C.A. (2017). Protein glycosylation in gastric and colorectal cancers: Toward cancer detection and targeted therapeutics. Cancer Lett..

[53] Trinchera M., Malagolini N., Chiricolo M., Santini D., Minni F., Caretti A., Dall’Olio F. (2011). The biosynthesis of the selectin-ligand sialyl Lewis x in colorectal cancer tissues is regulated by fucosyltransferase VI and can be inhibited by an RNA interference-based approach. Int. J. Biochem. Cell Biol..

[54] Balog C.I., Stavenhagen K., Fung W.L., Koeleman C.A., McDonnell L.A., Verhoeven A., Mesker W.E., Tollenaar R.A., Deelder A.M., Wuhrer M. (2012). N-glycosylation of Colorectal Cancer Tissues: A liquid chromatography and mass spectrometry-based investigation. Mol. Cell Proteom..

[55] Holst S., Stavenhagen K., Balog C.I., Koeleman C.A., McDonnell L.M., Mayboroda O.A., Verhoeven A., Mesker W.E., Tollenaar R.A., Deelder A.M. (2013). Investigations on aberrant glycosylation of glycosphingolipids in colorectal cancer tissues using liquid chromatography and matrix-assisted laser desorption time-of-flight mass spectrometry (MALDI-TOF-MS). Mol. Cell Proteom..

[56] Holst S., Deuss A.J., van Pelt G.W., van Vliet S.J., Garcia-Vallejo J.J., Koeleman C.A., Deelder A.M., Mesker W.E., Tollenaar R.A., Rombouts Y. (2016). N-glycosylation Profiling of Colorectal Cancer Cell Lines Reveals Association of Fucosylation with Differentiation and Caudal Type Homebox 1 (CDX1)/Villin mRNA Expression. Mol. Cell Proteom..

[57] Madunic K., Zhang T., Mayboroda O.A., Holst S., Stavenhagen K., Jin C., Karlsson N.G., Lageveen-Kammeijer G.S.M., Wuhrer M. (2021). Colorectal cancer cell lines show striking diversity of their O-glycome reflecting the cellular differentiation phenotype. Cell Mol. Life Sci..

[58] Kaprio T., Satomaa T., Heiskanen A., Hokke C.H., Deelder A.M., Mustonen H., Hagstrom J., Carpen O., Saarinen J., Haglund C. (2015). N-glycomic Profiling as a Tool to Separate Rectal Adenomas from Carcinomas. Mol. Cell Proteom..

[59] Kikuchi D., Saito M., Saito K., Watanabe Y., Matsumoto Y., Kanke Y., Onozawa H., Hayase S., Sakamoto W., Ishigame T. (2018). Upregulated solute carrier family 37 member 1 in colorectal cancer is associated with poor patient outcome and metastasis. Oncol. Lett..

[60] Yamadera M., Shinto E., Tsuda H., Kajiwara Y., Naito Y., Hase K., Yamamoto J., Ueno H. (2018). Sialyl Lewis(x) expression at the invasive front as a predictive marker of liver recurrence in stage II colorectal cancer. Oncol. Lett..

[61] Pothuraju R., Krishn S.R., Gautam S.K., Pai P., Ganguly K., Chaudhary S., Rachagani S., Kaur S., Batra S.K. (2020). Mechanistic and Functional Shades of Mucins and Associated Glycans in Colon Cancer. Cancers.

[62] Carvalho A.S., Harduin-Lepers A., Magalhaes A., Machado E., Mendes N., Costa L.T., Matthiesen R., Almeida R., Costa J., Reis C.A. (2010). Differential expression of a-2,3-sialyltransferases and a-1,3/4-fucosyltransferases regulates the levels of sialyl Lewis a and sialyl Lewis x in gastrointestinal carcinoma cells. Int. J. Biochem. Cell Biol..

[63] Perez-Garay M., Arteta B., Pages L., De Llorens R., de Bolos C., Vidal-Vanaclocha F., Peracaula R. (2010). a2,3-sialyltransferase ST3Gal III modulates pancreatic cancer cell motility and adhesion in vitro and enhances its metastatic potential in vivo. PLoS ONE.

[64] Dimitroff C.J., Pera P., Dall’Olio F., Matta K.L., Chandrasekaran E.V., Lau J.T., Bernacki R.J. (1999). Cell surface n-acetylneuraminic acid a2,3-galactoside-dependent intercellular adhesion of human colon cancer cells. Biochem. Biophys. Res. Commun..

[65] Gomes C., Osorio H., Pinto M.T., Campos D., Oliveira M.J., Reis C.A. (2013). Expression of ST3GAL4 leads to SLe^x^ expression and induces c-Met activation and an invasive phenotype in gastric carcinoma cells. PLoS ONE.

[66] Perez-Garay M., Arteta B., Llop E., Cobler L., Pages L., Ortiz R., Ferri M.J., de Bolos C., Figueras J., De Llorens R. (2013). a2,3-Sialyltransferase ST3Gal IV promotes migration and metastasis in pancreatic adenocarcinoma cells and tends to be highly expressed in pancreatic adenocarcinoma tissues. Int. J. Biochem. Cell Biol..

[67] Colomb F., Krzewinski-Recchi M.A., El Machhour F., Mensier E., Jaillard S., Steenackers A., Harduin-Lepers A., Lafitte J.J., Delannoy P., Groux-Degroote S. (2012). TNF regulates sialyl-Lewisx and 6-sulfo-sialyl-Lewisx expression in human lung through up-regulation of ST3GAL4 transcript isoform BX. Biochimie.

[68] Hiller K.M., Mayben J.P., Bendt K.M., Manousos G.A., Senger K., Cameron H.S., Weston B.W. (2000). Transfection of a1,3 fucosyltransferase antisense sequences impairs the proliferative and tumorigenic ability of human colon carcinoma cells. Mol. Carcinog..

[69] Weston B.W., Hiller K.M., Mayben J.P., Manousos G.A., Bendt K.M., Liu R., Cusack J.C. (1999). Expression of human a1,3 fucosyltransferase antisense sequences inhibits selectin-mediated adhesion and liver metastasis of colon carcinoma cells. Cancer Res..

[70] Pan S., Liu Y., Liu Q., Xiao Y., Liu B., Ren X., Qi X., Zhou H., Zeng C., Jia L. (2019). HOTAIR/miR-326/FUT6 axis facilitates colorectal cancer progression through regulating fucosylation of CD44 via PI3K/AKT/mTOR pathway. Biochim. Biophys. Acta Mol. Cell Res..

[71] Kudo T., Ikehara Y., Togayachi A., Morozumi K., Watanabe M., Nakamura M., Nishihara S., Narimatsu H. (1998). Up-regulation of a set of glycosyltransferase genes in human colorectal cancer. Lab. Investig..

[72] Holmes E.H., Hakomori S., Ostrander G.K. (1987). Synthesis of type 1 and 2 lacto series glycolipid antigens in human colonic adenocarcinoma and derived cell lines is due to activation of a normally unexpressed b1,3N-acetylglucosaminyltransferase. J. Biol. Chem..

[73] Marcos N.T., Magalhaes A., Ferreira B., Oliveira M.J., Carvalho A.S., Mendes N., Gilmartin T., Head S.R., Figueiredo C., David L. (2008). Helicobacter pylori induces b3GnT5 in human gastric cell lines, modulating expression of the SabA ligand sialyl-Lewis x. J. Clin. Investig..

[74] Lu C.H., Wu W.Y., Lai Y.J., Yang C.M., Yu L.C. (2014). Suppression of B3GNT7 gene expression in colon adenocarcinoma and its potential effect in the metastasis of colon cancer cells. Glycobiology.

[75] Shiozaki K., Yamaguchi K., Takahashi K., Moriya S., Miyagi T. (2011). Regulation of Sialyl Lewis Antigen Expression in Colon Cancer Cells by Sialidase NEU4. J. Biol. Chem..

[76] Izawa M., Kumamoto K., Mitsuoka C., Kanamori C., Kanamori A., Ohmori K., Ishida H., Nakamura S., Kurata-Miura K., Sasaki K. (2000). Expression of sialyl 6-sulfo Lewis X is inversely correlated with conventional sialyl Lewis X expression in human colorectal cancer. Cancer Res..

[77] Yusa A., Miyazaki K., Kimura N., Izawa M., Kannagi R. (2010). Epigenetic silencing of the sulfate transporter gene DTDST induces sialyl Lewisx expression and accelerates proliferation of colon cancer cells. Cancer Res..

[78] Miyazaki K., Ohmori K., Izawa M., Koike T., Kumamoto K., Furukawa K., Ando T., Kiso M., Yamaji T., Hashimoto Y. (2004). Loss of disialyl Lewis^a^ the ligand for lymphocyte inhibitory receptor sialic acid-binding immunoglobulin-like lectin-7 (Siglec-7) associated with increased sialyl Lewis ^a^ expression on human colon cancers. Cancer Res..

[79] Huang H.C., Chao C.C., Wu P.H., Chung H.Y., Lee H.Y., Suen C.S., Hwang M.J., Cai B.H., Kannagi R. (2019). Epigenetic silencing of the synthesis of immunosuppressive Siglec ligand glycans by NF-kappaB/EZH2/YY1 axis in early-stage colon cancers. Biochim. Biophys. Acta Gene Regul. Mech..

[80] Huang H.C., Cai B.H., Suen C.S., Lee H.Y., Hwang M.J., Liu F.T., Kannagi R. (2020). BGN/TLR4/NF-B Mediates Epigenetic Silencing of Immunosuppressive Siglec Ligands in Colon Cancer Cells. Cells.

[81] Kawamura Y.I., Kawashima R., Fukunaga R., Hirai K., Toyama-Sorimachi N., Tokuhara M., Shimizu T., Dohi T. (2005). Introduction of Sd^a^ carbohydrate antigen in gastrointestinal cancer cells eliminates selectin ligands and inhibits metastasis. Cancer Res..

[82] Capon C., Maes E., Michalski J.C., Leffler H., Kim Y.S. (2001). Sd^a^-antigen-like structures carried on core 3 are prominent features of glycans from the mucin of normal human descending colon. Biochem. J..

[83] Foxall C., Watson S.R., Dowbenko D., Fennie C., Lasky L.A., Kiso M., Hasegawa A., Asa D., Brandley B.K. (1992). The three members of the selectin receptor family recognize a common carbohydrate epitope, the sialyl Lewis^x^ oligosaccharide. J. Cell Biol..

[84] Liang J.X., Liang Y., Gao W. (2016). Clinicopathological and prognostic significance of sialyl Lewis X overexpression in patients with cancer: A meta-analysis. OncoTargets Ther..

[85] Nakamori S., Kameyama M., Imaoka S., Furukawa H., Ishikawa O., Sasaki Y., Izumi Y., Irimura T. (1997). Involvement of carbohydrate antigen sialyl Lewis^x^ in colorectal cancer metastasis. Dis. Colon Rectum.

[86] Terraneo L., Avagliano L., Caretti A., Bianciardi P., Tosi D., Bulfamante G.P., Samaja M., Trinchera M. (2013). Expression of carbohydrate-antigen sialyl-Lewis a on colon cancer cells promotes xenograft growth and angiogenesis in nude mice. Int. J. Biochem. Cell Biol..

[87] Guerrero P.E., Miro L., Wong B.S., Massaguer A., Martinez-Bosch N., Llorens R., Navarro P., Konstantopoulos K., Llop E., Peracaula R. (2020). Knockdown of a2,3-Sialyltransferases Impairs Pancreatic Cancer Cell Migration, Invasion and E-selectin-Dependent Adhesion. Int. J. Mol. Sci..

[88] Carrascal M.A., Silva M., Ramalho J.S., Pen C., Martins M., Pascoal C., Amaral C., Serrano I., Oliveira M.J., Sackstein R. (2018). Inhibition of fucosylation in human invasive ductal carcinoma reduces E-selectin ligand expression, cell proliferation, and ERK1/2 and p38 MAPK activation. Mol. Oncol..

[89] Yoshihama N., Yamaguchi K., Chigita S., Mine M., Abe M., Ishii K., Kobayashi Y., Akimoto N., Mori Y., Sugiura T. (2015). A Novel Function of CD82/KAI1 in Sialyl Lewis Antigen-Mediated Adhesion of Cancer Cells: Evidence for an Anti-Metastasis Effect by Down-Regulation of Sialyl Lewis Antigens. PLoS ONE.

[90] Kohler S., Ullrich S., Richter U., Schumacher U. (2010). E-/P-selectins and colon carcinoma metastasis: First in vivo evidence for their crucial role in a clinically relevant model of spontaneous metastasis formation in the lung. Br. J. Cancer.

[91] Gebauer F., Wicklein D., Stubke K., Nehmann N., Schmidt A., Salamon J., Peldschus K., Nentwich M.F., Adam G., Tolstonog G. (2013). Selectin binding is essential for peritoneal carcinomatosis in a xenograft model of human pancreatic adenocarcinoma in pfp--/rag2--mice. Gut.

[92] Stubke K., Wicklein D., Herich L., Schumacher U., Nehmann N. (2012). Selectin-deficiency reduces the number of spontaneous metastases in a xenograft model of human breast cancer. Cancer Lett..

[93] Esposito M., Mondal N., Greco T.M., Wei Y., Spadazzi C., Lin S.C., Zheng H., Cheung C., Magnani J.L., Lin S.H. (2019). Bone vascular niche E-selectin induces mesenchymal-epithelial transition and Wnt activation in cancer cells to promote bone metastasis. Nat. Cell Biol..

[94] Kelm M., Quiros M., Azcutia V., Boerner K., Cummings R.D., Nusrat A., Brazil J.C., Parkos C.A. (2020). Targeting epithelium-expressed sialyl Lewis glycans improves colonic mucosal wound healing and protects against colitis. JCI Insight.

[95] Bordon Y. (2015). Inflammation: Live long and prosper with Siglecs. Nat. Rev. Immunol..

[96] Deschepper F.M., Zoppi R., Pirro M., Hensbergen P.J., Dall’Olio F., Kotsias M., Gardner R.A., Spencer D.I.R., Videira P.A. (2020). L1CAM as an E-selectin Ligand in Colon Cancer. Int. J. Mol. Sci..

[97] Pucci M., Gomes F.I., Malagolini N., Ferracin M., Dall’Olio F. (2020). The Sd^a^ Synthase B4GALNT2 Reduces Malignancy and Stemness in Colon Cancer Cell Lines Independently of Sialyl Lewis X Inhibition. Int. J. Mol. Sci..

[98] Zhan L., Chen L., Chen Z. (2018). Knockdown of FUT3 disrupts the proliferation, migration, tumorigenesis and TGF-beta induced EMT in pancreatic cancer cells. Oncol. Lett..

[99] Kawamura Y.I., Adachi Y., Curiel D.T., Kawashima R., Kannagi R., Nishimoto N., Dohi T. (2014). Therapeutic adenoviral gene transfer of a glycosyltransferase for prevention of peritoneal dissemination and metastasis of gastric cancer. Cancer Gene Ther..

